# Decisions Informed by Computing Entities (DICE) to improve prognostic value of myocardial perfusion imaging: the NHLBI-sponsored Women's Ischemia Syndrome Evaluation (WISE) Study

**DOI:** 10.1186/1532-429X-14-S1-P6

**Published:** 2012-02-01

**Authors:** Mark Doyle, Gerald M Pohost, Leslee J Shaw, Sheryl F Kelsey, BD Johnson, William J Rogers, Geetha Rayarao, Barry L Sharaf, Carl J Pepine, Noel B Merz, Robert W Biederman

**Affiliations:** 1 Allegheny General Hospital, Pittsburgh, PA, USA

## Summary

Arithmatic computational modeling permits improved prognostic detection via a combinatorial or independent method.

## Background

Quantitative analysis can improve myocardial perfusion imaging (MPI) for prediction of prognosis. We have designed a novel approach, Decisions Informed by Computed Entities (DICE), to enhance the prognostic ability of MPI data to predict major adverse cardiovascular events (MACE) in women with suspected ischemic heart disease. We applied DICE to cardiovascular magnetic resonance imaging (CMRI) and Single Positron Computed Tomography (SPECT) MPI data sets in the Women’s Ischemia Syndrome Evaluation (WISE) Study.

## Methods

Women (n=228), mean age 59±11yrs, with symptoms suggestive of myocardial ischemia underwent MPI and cardiac function evaluation separately by CMRI and SPECT and were followed (40±17mo) for time to MACE (CV death, MI, and hospitalization for CHF). Abnormal perfusion defects were noted for each MPI modality (clinical reading). Cardiac regions were separately evaluated and at least one abnormal region was considered a positive study. The CMRI data were evaluated using qualitative (QLMR) and semi-quantitative (SQMR) approaches. Multiple linear regression models (DICE models) were generated, each predicting MPI status from one modality (e.g. CMRI) using data from a second modality (e.g. SPECT). Two DICE models were constructed from pairs of data sets and included variables such as end-systolic volume and myocardial wall thickness in addition to the clinical MPI reading.

## Results

MACE occurred in 29 women (13%). The percentage of MACE occurring in pts with abnormal perfusion was 43% using the average of the clinical MPI readings vs. 75% for the average of the DICE models (p<0.01). The average percentage of pts having abnormal perfusion was 27% for the clinical readings vs. 35% for the average of the DICE models (p=0.09). Increasing the number of variables in each DICE model increased the percentage of MACE captured.

## Conclusions

DICE modeling, incorporating the clinical MPI reading and cardiac variables derived from one imaging modality predicted the clinical MPI reading of a second modality while increasing the prognostic value as compared to just the clinical reading. The predictive power of the DICE model increases with the number of variables measured while simultaneously reducing noise and bias.

## Funding

NHLBI/Internal.

